# Pleistocene-dated biogeographic barriers drove divergence within the Australo-Papuan region in a sex-specific manner: an example in a widespread Australian songbird

**DOI:** 10.1038/s41437-019-0206-2

**Published:** 2019-03-15

**Authors:** Annika Mae Lamb, Anders Gonçalves da Silva, Leo Joseph, Paul Sunnucks, Alexandra Pavlova

**Affiliations:** 1 School of Biological Sciences, Monash University, Clayton Campus, Melbourne, VIC 3800 Australia; 2 School of BioSciences, The University of Melbourne, Parkville Campus, Melbourne, VIC 3010 Australia; 3 Microbial Diagnostic Unit Public Health Laboratory, The Peter Doherty for Infection and Immunity, The University of Melbourne, Melbourne, VIC 3010 Australia; 4 Australian National Wildlife Collection, CSIRO National Research Collections, GPO Box 1700, Canberra, ACT 2601 Australia

**Keywords:** Biogeography, Population genetics, Speciation, Phylogenetics

## Abstract

Understanding how environmental change has shaped species evolution can inform predictions of how future climate change might continue to do so. Research of widespread biological systems spanning multiple climates that have been subject to environmental change can yield generalizable inferences about the neutral and adaptive processes driving lineage divergence during periods of environmental change. We contribute to the growing body of multi-locus phylogeographic studies investigating the effect of Pleistocene climate change on species evolution by focusing on a widespread Australo-Papuan songbird with several mitochondrial lineages that diverged during the Pleistocene, the grey shrike-thrush (*Colluricincla harmonica*). We employed multi-locus phylogenetic, population genetic and coalescent analyses to (1) assess whether nuclear genetic diversity suggests a history congruent with that based on phenotypically defined subspecies ranges, mitochondrial clade boundaries and putative biogeographical barriers, (2) estimate genetic diversity within and genetic differentiation and gene flow among regional populations and (3) estimate population divergence times. The five currently recognized subspecies of grey shrike-thrush are genetically differentiated in nuclear and mitochondrial genomes, but connected by low levels of gene flow. Divergences among these populations are concordant with recognized historical biogeographical barriers and date to the Pleistocene. Discordance in the order of population divergence events based on mitochondrial and nuclear genomes suggests a history of sex-biased gene flow and/or mitochondrial introgression at secondary contacts. This study demonstrates that climate change can impact sexes with different dispersal biology in different ways. Incongruence between population and mitochondrial trees calls for a genome-wide investigation into dispersal, mitochondrial introgression and mitonuclear evolution.

## Introduction

The relationship between environmental change and species evolution is convoluted due to the plethora of ways in which species are impacted by and can respond to change. Understanding the population processes affected by environmental change is important for informing predictive models of species’ responses to future climate change. Climatic oscillations throughout the Pleistocene (~2.5–0.01 million years ago; Ma) impacted the evolution of biota across the globe through fragmentation, displacement and extinction (Hewitt 2004). Populations surviving glacial maxima in disconnected refugia often diverged under effects of different climatic and ecological pressures and genetic drift (Avise 1998, 2000). Australo-Papua, however, remained largely free from Pleistocene glaciation and instead experienced cycles of aridity and sea-level fluctuations (Barrows et al. 2002; Byrne et al. 2008).

Based on contemporary species’ ranges and taxonomic treatments, numerous putative historical biogeographical barriers, thought to reflect rising sea levels and expanded arid areas that formed during the Pleistocene, have been mapped across mainland Australia (Ford 1987; Keast 1961; Schodde and Mason 1999). Two contemporary marine barriers to gene flow, Bass Strait (in the South) and Torres Strait (in the North), formed and dissipated repeatedly throughout the Pleistocene and now separate mainland Australia from Tasmania and the island of New Guinea, respectively (Chivas et al. 2001; Lambeck and Chappell 2001). Speciation events across several of these biogeographical barriers support their role in shaping the evolution of Australian biota (Dolman and Joseph 2015, 2016; Jennings and Edwards 2005; Lee and Edwards 2008; Mellick et al. 2014; Toon et al. 2010).

The biological influence of some Australo-Papuan barriers, that is, how they limited migration and affected local adaptation and lineage divergence, remains to be fully explored with molecular data (e.g., Torres Strait—Kearns et al. 2011; Toon et al. 2017; Canning Barrier—Lamb et al. 2018; Nyári and Joseph 2013). Moreover, earlier phylogeographic investigations of putative Australo-Papuan barriers employed exclusively mitochondrial DNA (mtDNA) markers (Joseph and Omland 2009), which are prone to the effect of selection and do not always reflect population history (Ballard and Whitlock 2004; Morales et al. 2015; Toews and Brelsford 2012). Multi-locus tests of mitochondrial-based inferences using nuclear data are called for.

Pleistocene climate change impacted the evolution of different species in different ways, depending on their ecology (Bowman et al. 2010; Byrne et al. 2008). The biogeographical barriers drove the divergence of species at different times and to different extents depending on species’ gene flow limitations (Bowman et al. 2010; Dolman and Joseph 2012; Toon et al. 2010). Furthermore, arid-adapted species have expanded their ranges during Pleistocene glacial maxima, contrary to the majority of other species studied, which experienced range contractions (e.g., butcherbirds; Kearns et al. 2014). Different responses to climate change can result in different temporal patterns of divergence across a barrier and also lead to different geographic patterns of population differentiation (Bryant and Krosch 2016; Peñalba et al. 2017). Temporally and spatially concordant biogeographic patterns can therefore be expected only in ecologically similar sympatric species. Further, some barriers have formed, disappeared and reformed over time, such that disconnected populations have experienced multiple periods of secondary contact. In some cases of secondary contact, gene flow can homogenize genetic variation among populations (Joseph and Wilke 2006; Kearns et al. 2014), and in others, intrinsic barriers evolved during isolation (i.e., genomic incompatibilities) can drive evolution of pre-zygotic isolation and speciation (Sunnucks et al. 2017).

In addition to changing over time, climate varies greatly across the Australo-Papuan region. Climatic differences across the region may have further driven local adaptation and divergence within some species (Lamb et al. 2018). The dynamic spatio-temporal nature of Pleistocene population splits, periods of secondary contact and climatic variation has promoted phylogeographic complexity. Furthermore, putative barriers have not always been found to be concordant with intra- or interspecific nuclear and/or mitochondrial genetic divergence, sometimes despite their concordance with phenotypic divergence (Eldridge et al. 2014; Kearns et al. 2010). Because of these complexities, more multi-locus phylogeographic research on widespread species spanning multiple climates is needed. This will aid in achieving a comprehensive understanding of how Pleistocene climate change influenced the evolution of the biota throughout the Australo-Papuan region.

**Fig. 1:**
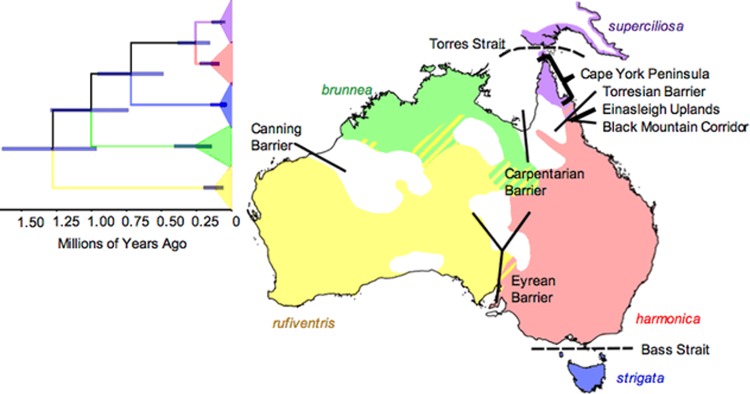
The ranges of the currently accepted grey shrike-thrush subspecies: blue—*strigata*, red—*harmonica*, purple—*superciliosa*, green—*brunnea* and yellow—*rufiventris*, and contemporary and putative historical biogeographic barriers and relevant geographic regions (Schodde and Mason 1999). The grey shrike-thrush mitochondrial ND2 tree topology adapted from Lamb et al. (2018) is also shown (same colours apply); here we considered the King Island and Tasmanian lineages as one because their ranges overlap

The grey shrike-thrush (*Colluricincla harmonica*) is an ideal species in which to investigate the complex evolutionary effects of Pleistocene climate: it is widespread and common throughout Australia and eastern coastal New Guinea and divergence among its mitochondrial clades has been dated to the Pleistocene (Higgins and Peter 2002; Lamb et al. 2018). Analysis of mitochondrial sequence data throughout the Australian range found clade boundaries to be concordant with phenotypically defined subspecies ranges (Lamb et al. 2018; Fig. 1). Further, Marki et al. (2018) found that within the New Guinean range of the species, DNA sequence data from two individuals from two different regions suggested the presence of two lineages, one that has, and one that has not diverged from the Cape York Peninsula populations of Australia. However, denser sampling of the region is required to test this observation. Eight putative historical biogeographical and contemporary barriers align with mitochondrial clade and subspecies boundaries within grey shrike-thrush and so could have affected its evolutionary history: the Carpentarian, Eyrean, Canning, and Torresian Barriers, Einasleigh Uplands, Black Mountain Corridor, and Bass and Torres Straits (Fig. 1). These barriers have primarily been inferred from morphological and molecular data on bird species and have been tested in a range of other taxa (see Appendix S1 for a brief review).

Here we use multi-locus phylogeography to test whether the barriers mentioned above have had different effects on population diversification and mitochondrial lineage divergence within the grey shrike-thrush. Specifically, we (1) test for congruence between patterns of nuclear genetic differentiation and those based on phenotypically defined subspecies ranges (Schodde and Mason 1999), mitochondrial clade boundaries and putative biogeographical barriers, (2) quantify genetic diversity within, and genetic differentiation and gene flow among regional populations, and (3) estimate population divergence times.

## Materials and methods

### Study species

Across its Australian range, five regional populations of grey shrike-thrush are currently recognized as subspecies based on morphometrics and plumage (epithets only hereafter for brevity: *rufiventris, brunnea, superciliosa, harmonica, strigata*; Fig. 1; Schodde and Mason 1999). New Guinean populations occur almost exclusively in Papua New Guinea (PNG); they are sometimes assigned to a separate subspecies (*tachycrypta*) but are mostly synonymized with *superciliosa* of Cape York Peninsula (Fig. 1; Beehler and Pratt 2016; Macdonald 1973; Schodde and Mason 1999).

### Samples, DNA extractions, DNA sequencing and length-variable marker genotyping

Lamb et al. (2018) obtained mitochondrial ND2 sequences for 170 grey shrike-thrush individuals sampled from the Australian range of the species. Here we extend that work and that of Marki et al. (2018) by denser sampling of New Guinean populations and screening of 20 loci in the nuclear genome, enabling new analyses and hypothesis tests. We sampled PNG grey shrike-thrush individuals from three isolated savannah areas: the Trans-Fly region of the Western Province of PNG and adjacent Indonesia and immediately north of Cape York Peninsula, the north coast of PNG in Oro Province, and the south coast of PNG in Central Province (see Appendix S2 in Supporting Information). The sex of the grey shrike-thrush individuals was inferred from phenotype and/or molecular sexing (Appendix 2). A Qiagen extraction kit was used to extract the genomic DNA from 19 PNG and Cape York Peninsula tissue samples from the Australian National Wildlife Collection (ANWC, CSIRO, Canberra), following the manufacturer’s instructions. These 19 individuals were sequenced for the ND2 locus following the approach in Pavlova et al. (2014), augmenting the total ND2 dataset to 189 sequences (Appendix S2). A subset of 69 individuals, representative of the entire range of the species, was further sequenced for one Z-linked and five autosomal intron loci (see Appendix S2 for a list of sequenced individuals and Appendix S3 in Supporting Information for amplification and sequencing details). The Phase 2.1 algorithm (Stephens and Donnelly 2003) implemented in DnaSP 6.11.01 (Rozas et al. 2017) was used to resolve the gametic phases of the nuclear intron sequences. For the Z-linked nuclear intron locus, only sequences from males were phased to account for hemizygosity of the Z-chromosome in females. Sequences containing double-indels could not be accurately aligned and phased and so were excluded from analyses. All sequenced loci were tested for significant deviations from linkage equilibria in each of the grey shrike-thrush populations using GENEPOP 4.2 (Raymond and Rousset 2004); for this analysis, populations were defined based on ND2 clade membership (see below) because of the tight correlation between ND2 clade and subspecies geographic boundaries. We further genotyped 170 individuals with sampling locations within Australia for 14 length-variable markers (Appendix S2): 11 microsatellite (BMC3, Cpi4, FhU2, FT25, HrU2, Pdo5, Ppi2, Ppm1, Ppm11, Ppm3, Ppm7) and 3 variable-length exon-primed-intron-crossing loci (23989, 4550s1, 24254s) that had previously been shown to be in linkage equilibria in grey shrike-thrush populations (Pavlova et al. 2012). Individuals from PNG were not included in these length-variable nuclear assays, because they were collected in the field after analyses of the microsatellite and variable-length exon-primed-intron-crossing dataset was complete.

**Fig. 2:**
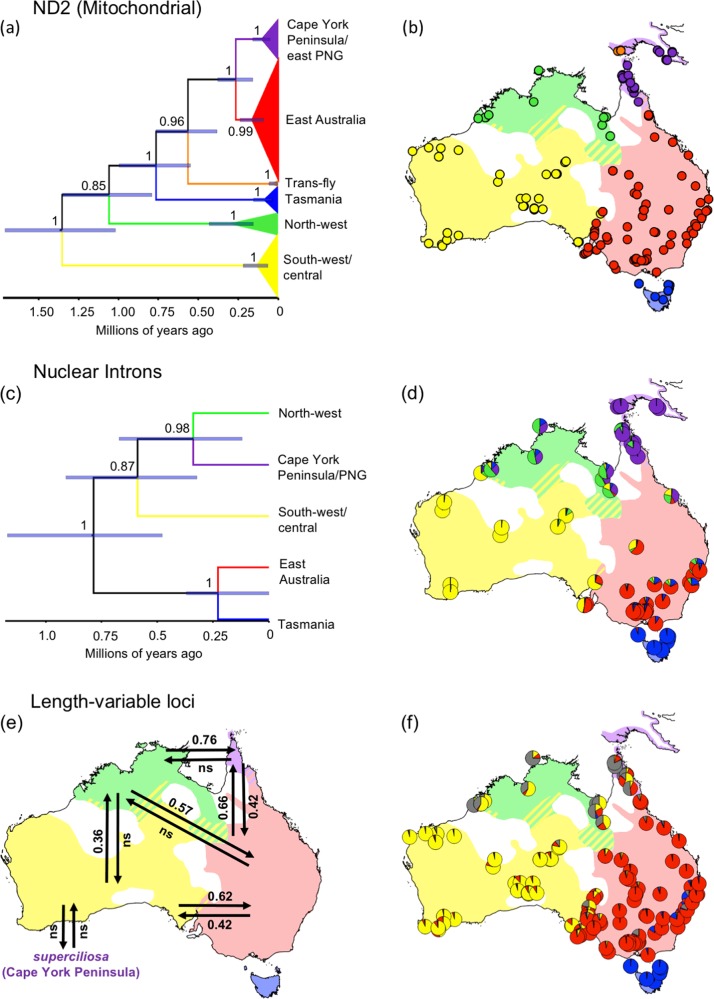
Individual membership to genetic clades, phylogenetic ND2 gene tree (**a**) and nuclear intron species tree (**c**) showing divergence among clades and gene flow between subspecies. Subspecies ranges are coloured as in Fig. 1. Mapped individuals in (**b**) are coloured by mitochondrial clade based on phylogenetic analysis of ND2 data. Pies in (**d**) and (**f**) represent genetic cluster membership probabilities identified by TESS analyses of the nuclear intron data (**d**) and length-variable marker data (**f**). Note that grey slices in the pies in (**f**) represent the genetic cluster shared by *brunnea* and *superciliosa* individuals. Map on (**e**) shows effective immigration rate estimates (in number of effective migrants per generation (confidence interval)) inferred by MIGRATE-N from the length-variable marker data. Where confidence intervals overlapped zero, effective immigration rates are shown as nonsignificant (ns)

### Assessing nuclear genetic structure

Nuclear genetic structure within the species was assessed using Bayesian clustering using tessellations in TESS 2.3.1 (Chen et al. 2007) to test whether putative biogeographical barriers, shown to be concordant with phenotypically defined subspecies boundaries and mitochondrial clade boundaries, are also concordant with nuclear genetic structure. Two analyses were run: one on length-variable marker genotypes, the other on nuclear intron sequence data (excluding the data for the sex-linked locus) in which the phased alleles at the sequenced introns were considered diploid genotypes. Individuals missing >20% of genotypes were excluded from analyses. A BYM model was implemented with a parameter of Dirichlet allele frequency model *D* = 1.0, a spatial interaction parameter *P* *=* 0.6 and a trend degree *T* = 2 (Durand et al. 2009). TESS was run assuming a range of cluster values (*K* from 2 to 10); for each *K*, 100 replicates of 30,000 burn-in sweeps followed by 100,000 sweeps were run. The calculated cluster probabilities of the 10 replicates with the lowest deviance information criterion (DIC) values of each *K* were averaged using CLUMPP 1.1.2 (Jakobsson and Rosenberg 2007). The most likely number of genetic clusters was identified based on run DIC values (see Appendix S4 in Supporting Information for details). To visualize the geographic arrangement of clusters for each nuclear dataset, we mapped memberships of each individual in genetic clusters over subspecies distributions (Schodde and Mason 1999) using Quantum GIS (qGIS 2.14.3) (qGIS Development QGIS Development Team 2017). Within-locus haplotypic relationships of the nuclear intron loci were analyzed using median-joining networks (Bandelt et al. 1999) constructed in PopArt (Leigh and Bryant 2015).

### Estimating divergence times among mitochondrial lineages and regional populations

Coalescent analyses were conducted to estimate the timing of mitochondrial lineage divergence and population divergence based on nuclear data to test for concordance between nuclear and mitochondrial evolution. Co-analysis of mitochondrial and nuclear DNA data using *BEAST within BEAST 2.4.7 (Bouckaert et al. 2014) was attempted but the parameters did not converge (low effective sample size (ESS) values and multimodal parameter distributions). Mitochondrial ND2 and nuclear intron sequence data were therefore analyzed separately.

A mitochondrial phylogeny was constructed from the ND2 sequences including individuals from PNG in BEAST 2.4.7 (building on Lamb et al. 2018). The Bayesian Information Criterion implemented in PartitionFinder 1.1.1 (Lanfear et al. 2012) identified the optimal partitioning scheme of the ND2 alignment to be by codon position and the ideal models of molecular evolution to be HKY + I for position 1, TrN93 + I for position 2 and HKY for position 3. BEAST analyses were run with unlinked substitution rates and clock models but linked trees for the three partitions. Trial 100 million generation runs of BEAST were conducted using each of the coalescent tree priors and compared. The divergence times within the trees derived from these trail runs were consistent, irrespective of which coalescent tree prior was implemented. A Bayesian Coalescent Skyline plot prior (Drummond et al. 2005), which allows for complicated demographic histories (Ho and Shapiro 2011) was applied for the final analysis and a random starting tree was used. Divergence times were calibrated using ND2 clock rates estimated from the Hawaiian honeycreeper system (Lerner et al. 2011), which represents a comparable case of recent divergence ( < 2 my) among the lineages of a passerine species: prior clock rates (mean of a lognormal distribution; range of 95% sampled rates) were set at 6.3 × 10^−3^ (5.6–7.2 × 10^−3^) substitutions per site per million years (s/s/my) for codon position one, 1.6 × 10^−3^ (1.3–2.0 × 10^−3^) s/s/my for position two and 5.8 10^−2^ (5.2–6.3 × 10^−2^) s/s/my for position 3. Four runs of 100 million generations were conducted, sampled every 10,000 generations and checked for convergence using Tracer 1.6.0 (Rambaut and Drummond 2007). The runs were combined and thinned 10,000-fold using LogCombiner 1.8.3 (Drummond and Rambaut 2007) after discarding the first 10% as burn-in. The resulting set of trees was summarized using Tree Annotator 1.8.3 (Drummond and Rambaut 2007) to create a Maximum Clade Credibility tree visualized using FigTree 1.4.2 (Rambaut 2014) and strongly supported clades with posterior probability ≥0.90 were considered distinct mitolineages.

To estimate time of population divergence, we used a species tree approach implemented in *BEAST within BEAST 2.4.7 using the sequencing data for the six nuclear intron loci. PartitionFinder was used to identify the best-fit model of molecular evolution for each locus: AB4: K80, DRD4: K80 + I + G, GAPDH11: K80 + I, MUSK-I4: HKY, RI2: K80, and TGFb2: K80 + I + G. A strict clock model was implemented and the following prior clock rates were set: 1.05 × 10^−3^ (0.38–2.1 × 10^−3^) s/s/my for AB4, DRD4, MUSK-I4, RI2 and TGFb2, and 1.2 × 10^−3^ (0.78–1.7 × 10^−3^) s/s/my for GAPDH11 (Lerner et al. 2011). The five nuclear genetic clusters identified for grey shrike-thrush based on TESS analysis of nuclear intron data were treated a priori as different species in this analysis (Appendix S4): Tasmania, east Australian, Cape York Peninsula and PNG, north-west and south-west/central. The Yule speciation model and continuous-linear and constant root population size model were applied to species trees (Heled and Drummond 2010). The analysis was run for four replicates of 100 million generations, and sampled every 10,000 generations. The replicate runs were combined, summarized and visualized to generate a species tree based on the nuclear intron data using the approach outlined above. It should be noted that *BEAST assumes that there is no gene flow among diverged lineages and so the presence of gene flow may have biased divergence time estimates towards more recent times.

### Regional population genetic differentiation, within-population genetic diversity and tests of selection or demographic changes in ND2

We estimated differentiation among regional populations of grey shrike-thrush and across putative biogeographical barriers in Arlequin 3.5.2.1 (Excoffier and Lischer 2010) using pairwise *F*_ST_ for the length-variable marker data and *Φ*_ST_ for the ND2 and nuclear intron sequence data. For these analyses, individuals were assigned to populations based on their locations relative to putative barriers and compared with tests for correlations between the putative barriers and population differentiation (Fig. 1). To account for multiple comparisons (*n* = 56), a *P*-value corrected using a modified false discovery rate procedure (Benjamini and Yekutieli 2001) of 0.011 was used to determine the significance of *F*_ST_ and *Φ*_ST_ values. Length-variable marker diversity of each population was estimated by calculating expected heterozygosity in Arlequin 3.5 (Excoffier and Lischer 2010), number of private alleles using GenAlEx (Peakall and Smouse 2006) and allelic richness in FSTAT (Goudet 2001). Sequence diversity was assessed by calculating the number of segregating sites, number of haplotypes, haplotype diversity and nucleotide diversity of the ND2 and nuclear intron sequence data in Arlequin. θ_π_, a mutation-scaled estimate of effective population size derived from number of pairwise differences, was further estimated in Arlequin for the nuclear intron sequence dataset. The diversity indices of the nuclear intron dataset were averaged among loci to give mean diversity indices. Diversity indices of the Cape York Peninsula regional population could not be calculated for GAPDH11, because all but one of the relevant sequences showed double-indels. Tajima’s *D* and Fu and Li’s *F* statistic were calculated from the ND2 sequences of each regional population to test for selection and/or demographic change in DnaSP (Rozas et al. 2017).

### Estimating gene flow among regional populations

Nuclear gene flow among the Australian grey shrike-thrush regional populations was estimated from length-variable markers using MIGRATE-N 3.6.11 (Beerli and Felsenstein 2001). MIGRATE-N is a Bayesian analysis that co-estimates historical (Kuhner 2009) mutation-scaled migration rates *M* = m/µ and effective population sizes θ = 4Neµ, where m—immigration rate, µ—mutation rate, Ne—effective population size and 4—scalar for diploid data. The number of effective migrants per generation moving from population A to population B can be estimated using (*M*_A→B_θ_B_)/4; where *M*_A→B_ is the mutation-scaled migration rate from A to B, and θ_B_ is the effective population size B. The lower and upper bounds of confidence intervals around the number of effective migrants per generation were estimated by inputting, respectively, the 2.5th and 97.5th percentile estimates of *M*_A→B_ and θ_B_ into the same equation. The Tasmanian regional population was excluded from this analysis due to its contemporary isolation (supported by strong and significant differentiation; see Results) and to prevent problematic over-parameterization of the model. Among the Australian mainland populations, 12 asymmetric migration parameters were estimated. The dataset was subsampled using a random sample size of 12 (the smallest sample size for a regional population) so that an equal number of individuals was analyzed from each regional population. Analyses were run with gamma-distributed priors on *M* (mean 0.5) and θ (mean 10). This low migration rate prior ensured that the Markov chains were started in regions of parameter space that are consistent with the clear mitochondrial genetic structure seen among regional populations of the species (Lamb et al. 2018). The Brownian motion approximation of the stepwise mutation model was implemented and Watterson’s approximations of relative mutation rates were estimated from the data. Markov chain Monte Carlo (MCMC) analyses were run with four heated chains of temperatures 1.0, 1.5, 3.0 and 1 × 10^6^ for 20 replicates that each had a 500 sample burn-in and an additional 500 sample chain. Convergence of the MCMC was confirmed by unimodal and approximately normal posterior distributions and acceptance ratios between 0.2 and 0.6.

## Results

### Nuclear genetic structure—TESS analyses

For length-variable marker data (collected only for Australian grey shrike-thrush individuals), four genetic clusters were identified (Appendix S4). Three of these clusters align with the geographic ranges of *strigata*, *harmonica* and *rufiventris* and boundaries of their respective mitochondrial clades: Tasmania, east Australian and south-west/central (Lamb et al. 2018) (Fig. 2f). The two northern Australian subspecies, *brunnea* and *superciliosa*, comprise the fourth genetic cluster (Fig. 2f).

For nuclear intron sequence data (collected for the Australian and PNG individuals), five genetic clusters were identified across the entire range of the species (Appendix S4) and each genetic cluster mainly mapped to one of the five subspecies (Fig. 2d; see Appendix S5 for nuclear intron haplotype networks). That is, a single genetic cluster mainly mapped to the distributions of the PNG and Cape York Peninsula populations.

### Mitochondrial gene tree

Each of the four BEAST runs converged and combined ESS values were >200 for all parameters. The splitting order and divergence dates of Australian mitolineages (Fig. 2a) were consistent with those of Lamb et al. (2018) (Fig. 1; see Appendix S6 for detailed phylogeny). Consistent with Marki et al. (2018), New Guinea was found to have two mitochondrial lineages. One lineage was indistinguishable from the Cape York Peninsula lineage and included individuals from the Oro and Central PNG Provinces. Another lineage was found to be a sister to the east Australian and Cape York Peninsula/east PNG lineages, from which it was estimated to have diverged 0.38–0.76 Ma, and included individuals from the Trans-Fly region.

### Nuclear intron species tree

The four *BEAST runs converged, and combined ESS values were >200 for all parameters. The topology of the species tree (Fig. 2c) did not match that of the ND2 gene tree (Fig. 2a). Within the species tree, the ancestor of the east Australian and Tasmanian regional populations was estimated to have diverged 0.47–1.15 Ma from the ancestor of the south-west/central, Cape York Peninsula/east PNG and north-west regional populations. The east Australian and Tasmanian regional populations were estimated to have diverged 0.11–0.39 Ma. The south-west/central regional population was estimated to have diverged 0.32–0.90 Ma from the ancestor of the Cape York Peninsula/PNG and north-west regional populations. The Cape York Peninsula/PNG and north-west regional populations were estimated to have diverged 0.16–0.59 Ma.

### Genetic differentiation and diversity and ND2 selection in regional populations

With one exception, regional grey shrike-thrush populations separated by contemporary or putative historical biogeographical barriers were significantly differentiated from each other at ND2, microsatellite and three or more nuclear intron loci (*P* < 0.011) (Table 1). The exception was that differentiation across the Torres Strait (between Australia and New Guinea) was not significant for nuclear intron sequences.

**Table 1:** Pairwise *F*_ST_ and *Φ*_ST_ values estimating differentiation across contemporary and putative historical biogeographical barriers between the populations of grey shrike-thrush inhabiting the regions either side of each barrier

Regions compared	Barrier	Nuclear intron *Φ*_ST_						Length-variable marker *F*_ST_	ND2 *Φ*_ST_
		AB4	DRD4	GAPDH	MUSK-I4	RI2	TGFb2		
East-Australia V Tasmania	Bass Strait	**0.49** (**<0.001**)	**0.57** (**<0.001**)	0.48 (**<**0.001)	**0.09** (**0.009**)	0.19 (0.027)	0.29 (**<**0.001)	**0.34** (**<0.001**)	**0.87** (**<0.001**)
East-Australia V Cape York Peninsula	Torresian	**0.51** (**<0.001**)	0.00 (0.387)	0.15 (0.288)	**0.93** (**<0.001**)	0.06 (0.153)	**0.10** (**<0.001**)	**0.07** (**<0.001**)	**0.66** (**<0.001**)
East-Australia V north-west	Carpentarian	**0.48** (**<0.001**)	**0.25** (**<0.001**)	0.00 (0.315)	**0.93** (**<0.001**)	0.04 (0.207)	**0.10** (**<0.001**)	**0.09** (**<0.001**)	**0.91** (**<0.001**)
East-Australia V south-west/central	Eyrean	**0.50** (**<0.001**)	**0.20** (**<0.001**)	**0.169** (**<0.001**)	**0.85** (**<0.001**)	**0.44** (**<0.001**)	**0.18** (**<0.001**)	**0.07** (**<0.001**)	**0.91** (**<0.001**)
Cape York Peninsula V PNG	Torres Strait	0.14 (0.216)	0.03 (0.126)	−0.27 (0.991)	0.00 (0.423)	0.05 (0.378)	0.10 (0.091)	NA	**0.19** (**<0.001**)
Cape York Peninsula V north-west	Carpentarian	0.24 (0.027)	**0.36** (**0.009**)	0.18 (0.108)	**0.68** (**<0.001**)	−0.08 (0.991)	0.10 (0.054)	**0.05** (**<0.001**)	**0.92** (**<0.001**)
North-west V south-west/central	Canning	0.09 (0.198)	**0.369** (**<0.001**)	−0.06 (0.68)	**0.94** (**<0.001**)	**0.67** (**<0.001)**	**0.46** (**<0.001**)	**0.08** (**<0.001**)	**0.94** (**<0.00**1)

Significant (*P*-value *<* 0.011) estimates are in bold. Length-variable marker data were not available for the Papua New Guinean (PNG) range of the species, so differentiation across the Torres Strait could not be estimated using this dataset (NA)

Relative to mainland Australian populations, the Tasmanian population was consistently found to have low genetic diversity in length-variable markers, mitochondrial ND2 and nuclear intron sequences (Table 2). The PNG population also had relatively low genetic diversity in nuclear intron sequences (Table 2), but harboured two mitochondrial ND2 lineages with relatively high combined nucleotide diversity (Table 2). Effective population sizes derived from the length-variable marker dataset were similar among mainland populations (Table 2). Relative to the mainland populations, the effective population sizes of the Tasmanian and PNG populations estimated using nuclear intron sequences were low (Table 2).

**Table 2:** Estimates of genetic diversity for the regional populations of *C. harmonica* and results of tests of selection/demographic change (Tajima’s *D* and Fu and Li’s *F* statistic estimates) in ND2

Dataset	Regional population	Tasmanian	East Australia	Cape York Peninsula	Papua New Guinea	North-west	South-west/central
Length-variable markers	*N* inds	18	83	12	–	15	42
	% Polymorphic loci	35.7	100.0	100.0	–	78.6	85.7
	*N* alleles/locus	2.64	9.50	5.64	–	5.93	7.71
	*N* private alleles/locus	0.29	1.93	0.29	–	0.57	1.00
	AR	2.93	5.52	5.64	–	5.61	5.38
	He (sd)	0.23 (0.13)	0.57 (0.29)	0.55 (0.29)	–	0.53 (0.28)	0.52 (0.27)
	θ	–	4.29	4.90	–	4.29	4.26
ND2	*N* inds	18	84	15	16	15	41
	S	5	80	12	30	23	31
	H	6	37	9	6	12	25
	Hd	0.719	0.903	0.848	0.808	0.971	0.955
	π	0.0015	0.004	0.0028	0.010	0.0042	0.003
	Tajima’s *D* (*P*-value)	0.151 (>0.10)	−2.191 (<0.01)	−0.860 (>0.10)	0.771 (>0.10)	−1.576 (>0.10)	−1.934 (<0.05)
	Fu and Li’s *F* (*P*-value)	−0.252 (>0.10)	−3.503 (<0.02)	−1.209 (>0.10)	1.457 (>0.05)	−1.962 (>0.10)	−2.812 (<0.05)
Nuclear introns	*N* inds mean (min–max)	6.2 (2–7)	32.2 (29–33)	5.7 (1–7)	7.7 (6–8)	6.2 (2–7)	7.5 (6–8)
	S mean (min–max)	2.7 (0–9)	13.0 (2–29)	6.2 (1–16)	3.7 (0–12)	6.3 (1–18)	5 (0–9)
	H mean (min–max)	1.8 (1–3)	15.2 (3–32)	4.4 (2–9)	2.7 (1–5)	4.7 (2–10)	5.2 (1–9)
	Hd mean (min–max)	0.263 (0–0.833)	0.703 (0.461–0.966)	0.559 (0.20–0.923)	0.296 (0–0.525)	0.621 (0.264–1)	0.550 (0–0.9242)
	π mean (min–max)	0.0026 (0–0.0123)	0.0053 (0.0009–0.0111)	0.0035 (0.0007–0.0095)	0.0020 (0–0.0039)	0.0046 (0.0009–0.0118)	0.0031 (0–0.0101)
	θ_π_ (min–max)	0.93 (0–3.83)	2.39 (0.48–5.45)	1.90 (0.2–5.63)	0.87 (0–2.32)	2.04 (0.26–5.49)	1.33 (0–3.14)

The number of individuals analyzed for each dataset (*N* inds); percentage polymorphic loci, number of alleles per locus, number of private alleles per locus, allelic richness (AR) and heterozygosity (He) of the length-variable marker data; and the number of segregating sites (S), number of haplotypes (H), haplotype diversity (Hd) and nucleotide diversity (π) of sequence data are listed. Mutation-scaled estimates of effective population size calculated for the length-variable marker (θ) and nuclear intron (θ_π_) data are also listed

Tajima’s *D* and Fu and Li’s *F* statistic estimates were significantly negative for the ND2 sequences of the south-west/central and east Australian regional populations (Table 2). Estimates for all other regional populations were nonsignificant (Table 2).

### Nuclear gene flow among regional populations

Low levels of nuclear gene flow (mean effective population immigration rates <1 migrant per generation) were detected among the grey shrike-thrush regional populations (Fig. 2e; Appendix S7 in Supporting Information). Unidirectional gene flow was detected from north-west Australia to Cape York Peninsula and to east Australia, and from the south-west/central to the north-west regional populations (Fig. 2e). Bidirectional gene flow was detected between the Cape York Peninsula and east Australian and between east Australian and south-west/central populations (Fig. 2e). Gene flow in five other directions did not differ significantly from 0 (confidence intervals overlapped with 0; Appendix S7).

## Discussion

In using nuclear and mitochondrial genetic data to assess the roles of biogeographic barriers in the evolution of a widespread Australo-Papuan songbird, the grey shrike-thrush, we have generated four key findings. First, the geographic arrangement of nuclear genetic diversity was broadly concordant with mitochondrial genetic diversity, phenotypically defined subspecies ranges and contemporary or putative historical biogeographical barriers. Two exceptions to this were that the Trans-Fly population and the Cape York Peninsula/east PNG populations are distinguishable based on mitochondrial but not nuclear sequence data, and the Cape York Peninsula and north-west populations are distinguishable based on mitochondrial data and cluster analysis of the nuclear intron sequence data, but not cluster analysis of the microsatellite data. Second, genetic differentiation across the implicated barriers was significant for mitochondrial, microsatellite and some nuclear intron data and dated to the Pleistocene (0.01–2.58 Ma). There was one exception to this: nuclear genetic differentiation was not significant across the Torres Strait. Third, the order of population divergence inferred from mtDNA and nuclear DNA (nDNA) is not consistent, suggesting a history of sex-biased gene flow and/or mitochondrial introgression. Finally, parapatric regional populations appear to be connected by low levels of gene flow (<1 migrant per generation).

### Discrepancies between mitochondrial ND2 and nuclear intron evolution

The timing and order of divergence events inferred from mtDNA and nDNA are not consistent. Unusually for songbirds, the grey shrike-thrush has male-biased dispersal (Pavlova et al. 2012), which can slow down population divergence at nuclear genes while maintaining divergence of the mitochondrial genome (Melnick and Hoelzer 1992). Therefore, a low level of nuclear gene flow among the parapatric Australian regional populations, which harbour different mitochondrial lineages, could be mediated by males. Differing levels of male-mediated gene flow among population pairs and female-mediated mitochondrial introgression can influence the order of apparent divergence events on nuclear and mitochondrial genomes (Ballard and Whitlock 2004; Havird and Sloan 2016; Morales et al. 2017; Toews and Brelsford 2012). Lineage sorting is expected to be slower for nuclear autosomal loci than for the mitochondrial genome because of a larger effective population size (Charlesworth 2002). Relatively rapid lineage sorting at mitochondrial loci may have resulted in the more ancient divergence among lineages at mitochondrial compared with nuclear loci seen across some barriers. However, different rates of lineage sorting cannot explain the different order of splitting events in the mitochondrial gene tree and nuclear intron species tree. For example, in two cases, sister groups that are well-supported on nuclear data, North-west and Cape York Peninsula/PNG, diverged 0.16–0.59 Ma, and East Australia and Tasmania, diverged 0.11–0.39 Ma, are interspersed with other lineages on the mitochondrial tree, with divergence dating to 0.79–1.35 Ma and 0.55–1.00 Ma, respectively (Fig. 2). Even if the mutation rate priors applied for the coalescent analyses are not appropriate for one or multiple loci, this would have resulted in consistent differences in divergence times between the mitochondrial gene and nuclear intron species trees. Thus, neither different rates of lineage sorting nor inappropriate mutation rate priors, alone or together, can explain different tree topologies derived from mitochondrial and nuclear markers. Different combinations of purifying, positive and frequency-dependent natural selection, however, can have complex effects on phylogenies (Edwards 2009). Significantly negative Tajima’s *D* and Fu and Li’s *F* statistic estimates for the mitochondrial locus indicate a selective sweep or purifying selection and/or recent population expansion in the east Australian and south-west/central regional populations. Furthermore, mitochondrial sequence variation within grey shrike-thrush has previously been associated with aridity, and a candidate amino-acid target of positive selection was identified in ND6 for the south-west/central mitolineage through comparative analysis (Lamb et al. 2018). It is possible that climate-driven selection drove adaptive mitochondrial introgression, resulting in discrepancies between mitochondrial and nuclear DNA evolution, specific cases of which we discuss below.

### Evidence of biogeographical barriers driving divergence during the Pleistocene

The Carpentarian Barrier is concordant with significant mitochondrial and nuclear DNA differentiation dated to the Pleistocene between the north-west and Cape York Peninsula and north-west and east Australian forms of grey shrike-thrush. Notably, clustering analysis of length-variable markers did not reveal a phylogeographic break concordant with the Carpentarian barrier between the north-west and Cape York Peninsula populations. The length-variable markers in the dataset are mainly microsatellite loci and so this is contrary to the common view that microsatellites provide greater genetic resolution than sequence data because of their relatively high mutation rate; this could be due to homoplasy resulting from limit to size changes (see Coates et al. 2009 for explanatory mechanisms). Further, mtDNA divergence between the north-west and Cape York Peninsula/east PNG lineages was estimated to be relatively ancient compared with nuclear DNA divergence. We detected nuclear gene flow from the north-west to Cape York Peninsula population. Male-mediated dispersal via the land bridges that connected northern Australia and the island of New Guinea prior to the last marine transgression (9.7 ka) may have been sufficient to overcome some of the effect of the Carpentarian barrier on nuclear differentiation (Chivas et al. 2001; Torgersen et al. 1988; counter example in Eldridge et al. 2014). We infer that the Carpentarian Barrier drove vicariance-based phylogeographic structure of the grey shrike-thrush as seen in many, but not all, relevant species studied to date (Eldridge et al. 2014; Jennings and Edwards 2005; Joseph et al. 2011; Kearns et al. 2010, 2011; Lee and Edwards 2008; Potter et al. 2012; Schweizer et al. 2013). This, together with the variable timing of divergence across the Carpentarian barrier among species, indicates it may have had species-specific effects on divergence perhaps relating to dispersal ability, habitat preference and overall geographical range (Jennings and Edwards 2005; Lee and Edwards 2008; Schweizer et al. 2013; Toon et al. 2010).

The Eyrean Barrier is concordant with the substantial phylogeographic structure seen in nuclear and mitochondrial data between the east Australian and south-west/central regional forms of grey shrike-thrush. This barrier also fits Pleistocene phylogeographic breaks within a number of arid-adapted birds (Dolman and Joseph 2015; Joseph and Wilke 2006; Kearns et al. 2009; McElroy et al. 2018; see Alpers et al. 2016 and Neaves et al. 2012 for mammalian examples). In this study, divergence across the Eyrean Barrier dated to early to mid-Pleistocene (0.47–1.15 Ma) based on nuclear sequence data and early Pleistocene (1.02–1.71 Ma) based on mitochondrial sequence data. Bidirectional gene flow was detected across the Eyrean Barrier despite mitochondrial divergence, suggesting that male-biased dispersal might be operating. In addition, the east Australian and south-west/central regional populations have relatively high genetic diversity and effective population sizes, suggesting that the effect of drift in these populations is low. Thus, male-biased gene flow and/or a low level of genetic drift might be responsible for the more recent inference of divergence from nuclear intron data compared with mitochondrial data. Pleistocene-dated divergence times are consistent with the estimates for two sister species of quail-thrush (*Cinclosoma* spp.) and diversity within the mulga parrot (*Psephotellus varius*) (Dolman and Joseph 2016; McElroy et al. 2018). We infer that the Eyrean Barrier drove vicariance-based phylogeographic structure of the grey shrike-thrush as inferred for many other species.

Three barriers, the Torresian Barrier, the Black Mountain Corridor and Einasleigh Uplands, are concordant with phylogeographic structure between east Australian and Cape York Peninsula/east PNG regional forms of the grey shrike-thrush. This pattern is significant for nuclear and mitochondrial genetic markers, although relatively recent mitochondrial divergence was observed. This can be explained by vicariance, followed by secondary contact and mitochondrial introgression. Other studies show extreme variation in the time of divergence across these barriers among species (see Bryant and Krosch 2016 and examples therein); divergence within a genus of evergreen trees (*Elaeocarpus*) dates to 0.04–0.18 Ma while divergence within a genus of earthworm (*Terrisswalkerius*) dates to 31–84 Ma (Mellick et al. 2014; Moreau et al. 2015). Together with evidence of secondary contact within some relevant taxa, this indicates that multiple vicariance events have occurred across these three barriers that have had taxon-specific effects on divergence (Peñalba et al. 2017).

The Canning Barrier is reasonably inferred here as having been the driver of the mitochondrial and nuclear Pleistocene-dated phylogeographic structure between the south-west/central and north-west regional populations of grey shrike-thrush. This adds to the growing body of molecular data affirming this barrier as a Pleistocene driver of divergence (Lamb et al. 2018; Nyári and Joseph 2013). An ND6 amino acid may be evolving under positive selection in the south-west/central populations (Lamb et al. 2018), which may have further promoted divergence between the south-west/central and north-west populations.

Divergence across Bass Strait between east Australian and Tasmanian populations of grey shrike-thrush dates to 0.55–1.00 Ma based on ND2 diversity and 0.11–0.39 Ma based on nuclear intron diversity. These estimates predate the Last Glacial Maximum (LGM, ~21 ka). This suggests that gene flow was limited between these two regions despite LGM land bridges connecting them, as has been observed for the butterfly species *Heteronympha merope* (Norgate et al. 2009). Marshy habitat in exposed areas during the LGM (Lambeck and Chappell 2001) would have been unsuitable for grey shrike-thrush. Male-biased gene flow may explain more recent divergence across Bass Strait of nuclear intron lineages compared with ND2 lineages. Slow lineage sorting of nuclear DNA is less likely here since the Tasmanian population, having relatively low effective population size and low genetic diversity, is expected to experience higher rates of genetic drift.

The few molecular studies of trans-Torres Strait species have revealed a range of patterns in historical and contemporary gene flow among regions of north Australia and the island of New Guinea (Christidis et al. 1988; Edwards 1993; Kearns et al. 2011, 2014; Roshier et al. 2012; Toon et al. 2017). Cape York Peninsula and PNG populations of grey shrike-thrush are indistinguishable based on nuclear markers. This accords with their current classification as *C. h. superciliosa* and suggests they maintained male-mediated gene flow during the LGM across then-exposed savannah woodlands and grasslands (Nix and Kalma 1972; Williams et al. 2009). Grey shrike-thrush currently inhabit savannah areas within PNG and relatively shallow divergence across Torres Strait has been repeatedly observed in savannah-adapted species (Keighley et al. 2019; Murphy et al. 2007; Toon et al. 2017; Williams et al. 2008; Wüster et al. 2005) compared with deeper divergence observed in mesic closed forest-adapted species (Joseph et al. 2001; Kearns et al. 2011; Krajewski et al. 2004; Macqueen et al. 2011, 2010; Norman et al. 2007; Rawlings and Donnellan 2003; Zwiers et al. 2008). A mitochondrial lineage unique to the Trans-Fly was estimated to have diverged from mainland Australian and east PNG lineages 0.38–0.76 Ma. Distinct, albeit varyingly so, lineages have been identified within the Trans-Fly region for the eastern brown snake, the Australian magpie and the palm cockatoo (Murphy et al. 2007; Toon et al. 2017; Williams et al. 2008). The savannahs of the Trans-Fly and Central Province are separated by a markedly different zone of the Southern Lowlands of the Gulf Province with relatively high rainfall (Nix and Kalma 1972; Shearman and Bryan 2011). This zone may have acted as a driver of endemism within the Trans-Fly (Beehler and Pratt 2016; Heinsohn and Hope 2006; Kearns et al. 2011; Schodde 2006). Populations of grey shrike-thrush from the Oro and Central Provinces are indistinguishable based on nuclear and mtDNA. Though latitudinally separated by New Guinea’s central Cordillera, they may have experienced recent and current connectivity via a ring of lowland forest that surrounds the Cordillera (Dumbacher and Fleischer 2001; Kearns et al. 2011).

### Inferences about species evolution and taxonomy

The evolutionary history of the grey shrike-thrush appears more complex than a simple case of vicariant divergence; it has involved instances of discordant evolution of mitochondrial and nuclear DNA, asymmetric and sex-biased gene flow and apparent mitochondrial introgression. The five currently recognized subspecies of the grey shrike-thrush (New Guinean populations included in *C. h. superciliosa*) comprise five geographically and phylogenetically discrete clades for nuclear and mtDNA. Marki et al. (2018) identified deep divergence among the grey shrike-thrush lineages that could indicate multiple species. Compared with the rufous shrike-thrush *C. megarhyncha*, a closely related but non-sister species, the grey shrike-thrush displays an intermediate level of intra-specific divergence: the clades of *C. megarhyncha* are much older and likely represent different species, while *C. tenebrosa*, despite being widely sampled, showed little genetic variation (Marki et al. 2018). Significant genetic differentiation, Pleistocene-dated divergence, low gene flow and few discernable phenotypic differences among the Australian mainland subspecies place the species firmly in the grey zone of speciation, i.e., different species concepts disagree on placement of species limits (De Queiroz 2007; Roux et al. 2016). It is particularly difficult to infer the trajectory of the Australian regional populations. The putative barriers implicated in their divergence are now concordant with hybrid zones between them. The Australian regional populations may have experienced isolation during the Pleistocene and could now be experiencing secondary contact, where gene flow may be either promoting uniformity or being restricted by reproductive incompatibilities (Ottenburghs et al. 2017). Testing for the presence of intrinsic genomic incompatibilities using genome-wide data may indicate whether there are barrier loci between the regional populations preventing their admixture. In addition, an analysis incorporating estimates of divergence, gene flow and Ne from hundreds of genome-wide markers (Hey 2009) is required to clarify the evolutionary history of grey shrike-thrush populations and to determine where they stand on the speciation continuum.

## Conclusions

Pleistocene climate change has shaped the evolution of the grey shrike-thrush as it has for a diverse suite of other species. Cycles of marine transgressions and regressions and expansions and contractions of arid zones have driven divergence and sex-biased gene flow within the species. This system demonstrates that climate change can have sex-specific effects on evolution of species with different dispersal biology of sexes and this in turn has implications for the future of this and other species in our changing world. Incongruence between population and mitochondrial trees in this and other studies should trigger a genome-wide investigation into sex-biased population processes including dispersal, mitochondrial introgression and mitonuclear evolution.

### Data archiving

Sequence data have been submitted to GenBank: accession numbers MH316208–MH316549 and MH472639–MH472641. Sequences for the aldolase B intron 4 locus (<200 nucleotides in length) are included in Supporting Information S2. Length-variable marker genotypes are also included in Supporting Information S2.

## Supplementary information

Appendix S1

Appendix S2

Appendix S3

Appendix S4

Appendix S5

Appendix S6

Appendix S7
