# Supplementary material for: Pleistocene-dated biogeographic barriers drove divergence within the Australo-Papuan region in a sex-specific manner: an example in a widespread Australian songbird
Source: Heredity (Edinb). 2019 Mar 15;123(5):608–21. doi: 10.1038/s41437-019-0206-2 (PMC6972870; doi:10.1038/s41437-019-0206-2)
Supplement: Supplementary file 1 — Appendix S1 [file 41437_2019_206_MOESM1_ESM.doc]

**Appendix S1:** Brief review of implicated biogeographical barriers

The Carpentarian Barrier limited trans-Torresian migration during the Pleistocene and has been associated with genetic divergence within a number of Australian animals (Eldridge, Potter, Johnson, & Ritchie, 2014; Jennings & Edwards, 2005; Joseph, Zeriga, Adcock, & Langmore, 2011; Kearns, Joseph, Omland, & Cook, 2011; Lee & Edwards, 2008; Potter, Eldridge, Taggart, & Cooper, 2012; Schweizer, Guentert, & Hertwig, 2013). The timing of divergence across the Carpentarian Barrier is highly variable among species, spanning the early Pliocene through to the early Pleistocene (Lee & Edwards, 2008; Toon, Hughes, & Joseph, 2010).

The Eyrean Barrier represents the ephemeral lakes that recurrently formed and the extreme aridity that existed within the region throughout the Pleistocene (Joseph & Wilke, 2006). This barrier appears to have driven the evolution of numerous avian species (Dolman & Joseph, 2012; Dolman & Joseph, 2015, 2016; Joseph & Wilke, 2006; Kearns, Joseph, Edwards, & Double, 2009; Toon et al., 2010). Divergence across the Eyrean barrier has been dated to the mid-Pleistocene between two sister species of quail-thrush and to the late-Pleistocene in the mulga parrot (Dolman & Joseph, 2016; McElroy, Beattie, Symonds, & Joseph, 2017).

The Torresian and Black Mountain Corridor barriers represent regions of dry habitat that divide the Wet Topics that are thought to have been more arid during dry periods of the Pleistocene (Nicholls & Austin, 2005; Peñalba, Mason, Schodde, Moritz, & Joseph, 2017). The region between these two barriers encompasses a section of the Einasleigh Uplands; a part of the Great Dividing Range that separates the central coastal Queensland and Cape York Peninsula lowlands (Peñalba et al., 2017). The Torresian and Black Mountain Corridor barriers and the Einasleigh Uplands are situated between the Cape York Peninsula and east Australia regional populations of grey shrike-thrush such that their individual effects on species divergence cannot be distinguished here. Diverse animal and plant species display molecular and phenotypic divergence across the Torresian and Black Mountain Corridor barriers and the Einasleigh Uplands (Dolman & Moritz, 2006; Edwards & Melville, 2010; Hoskin et al., 2011; Joseph, Moritz, & Hugall, 1995; Kearns et al., 2011; Mellick, Wilson, & Rossetto, 2014; Moreau, Hugall, McDonald, Jamieson, & Moritz, 2015; Nicholls & Austin, 2005; Peñalba et al., 2017; Schneider, Cunningham, & Moritz, 1998; Toon et al., 2010). There is extreme variation in divergence estimates across these barriers among taxa: divergence within a genus of evergreen trees (*Elaeocarpus*) dates to 0.04 – 0.18 Ma while divergence within a genus of earthworm (*Terrisswalkerius*) dates to 31-84 Ma

The Canning Barrier has been much more rarely subjected to molecular testing than the Carpentarian, Eyrean, Torresian and Black Mountain Corridor barriers (Lamb et al., 2018; Nyári & Joseph, 2013). It is an arid barrier that has impacted the divergence between Kimberley and Pilbara populations of mangrove-specialised birds (Nyári & Joseph, 2013) and it is concordant with mid-Pleistocene phylogeographic breaks in two other widespread bird species (Lamb et al., 2018).

The Bass and Torres Straits, respectively, separate Tasmanian from mainland Australia populations and Cape York Peninsula from New Guinea. Tasmania (14 ka) and Papua New Guinea (9.7 ka), have been disconnected from the mainland since the last glacial maxima (LGM, ~21 ka) (Barrows, Stone, Fifield, & Cresswell, 2002; Chivas et al., 2001; Lambeck & Chappell, 2001).

**References**

Barrows, T. T., Stone, J. O., Fifield, L. K., & Cresswell, R. G. (2002). The timing of the last glacial maximum in Australia. *Quaternary Science Reviews, 21*(1), 159-173.

Chivas, A. R., Garcı́a, A., van der Kaars, S., Couapel, M. J., Holt, S., Reeves, J. M., . . . Banerjee, D. (2001). Sea-level and environmental changes since the last interglacial in the Gulf of Carpentaria, Australia: an overview. *Quaternary International, 83*, 19-46.

Dolman, G., & Joseph, L. (2012). A species assemblage approach to comparative phylogeography of birds in southern Australia. *Ecology and evolution, 2*(2), 354-369. doi:10.1002/ece3.87

Dolman, G., & Joseph, L. (2015). Evolutionary history of birds across southern Australia: structure, history and taxonomic implications of mitochondrial DNA diversity in an ecologically diverse suite of species. *Emu, 115*(1), 35-48.

Dolman, G., & Joseph, L. (2016). Multi-locus sequence data illuminate demographic drivers of Pleistocene speciation in semi-arid southern Australian birds (Cinclosoma spp.). *BMC evolutionary biology, 16*(1), 226.

Dolman, G., & Moritz, C. (2006). A multilocus perspective on refugial isolation and divergence in rainforest skinks (Carlia). *Evolution, 60*(3), 573-582.

Edwards, D. L., & Melville, J. (2010). Phylogeographic analysis detects congruent biogeographic patterns between a woodland agamid and Australian wet tropics taxa despite disparate evolutionary trajectories. *Journal of Biogeography, 37*(8), 1543-1556.

Eldridge, M. D., Potter, S., Johnson, C. N., & Ritchie, E. G. (2014). Differing impact of a major biogeographic barrier on genetic structure in two large kangaroos from the monsoon tropics of Northern Australia. *Ecology and evolution, 4*(5), 554-567.

Hoskin, C. J., Tonione, M., Higgie, M., MacKenzie, J. B., Williams, S. E., VanDerWal, J., & Moritz, C. (2011). Persistence in peripheral refugia promotes phenotypic divergence and speciation in a rainforest frog. *The American Naturalist, 178*(5), 561-578.

Jennings, W. B., & Edwards, S. V. (2005). Speciational history of Australian grass finches (Poephila) inferred from thirty gene trees. *Evolution, 59*(9), 2033-2047.

Joseph, L., Moritz, C., & Hugall, A. (1995). Molecular support for vicariance as a source of diversity in rainforest. *Proceedings of the Royal Society of London B: Biological Sciences, 260*(1358), 177-182.

Joseph, L., & Wilke, T. (2006). Molecular resolution of population history, systematics and historical biogeography of the Australian ringneck parrots Barnardius: are we there yet? *Emu, 106*(1), 49-62.

Joseph, L., Zeriga, T., Adcock, G. J., & Langmore, N. E. (2011). Phylogeography and taxonomy of the little bronze-cuckoo (Chalcites minutillus) in Australia’s monsoon tropics. *Emu, 111*(2), 113-119.

Kearns, A. M., Joseph, L., Edwards, S. V., & Double, M. C. (2009). Inferring the phylogeography and evolutionary history of the splendid fairy‐wren Malurus splendens from mitochondrial DNA and spectrophotometry. *Journal of Avian Biology, 40*(1), 7-17.

Kearns, A. M., Joseph, L., Omland, K. E., & Cook, L. G. (2011). Testing the effect of transient Plio-Pleistocene barriers in monsoonal Australo-Papua: did mangrove habitats maintain genetic connectivity in the Black Butcherbird? *Molecular Ecology, 20*(23), 5042-5059. doi:10.1111/j.1365-294X.2011.05330.x

Lamb, A. M., Gan, H. M., Greening, C., Joseph, L., Lee, Y. P., Moran-Ordonez, A., . . . Pavlova, A. (2018). Climate-driven mitochondrial selection: A test in Australian songbirds. *Mol Ecol*. doi:10.1111/mec.14488

Lambeck, K., & Chappell, J. (2001). Sea level change through the last glacial cycle. *Science, 292*(5517), 679-686.

Lee, J. Y., & Edwards, S. V. (2008). Divergence across Australia's Carpentarian barrier: statistical phylogeography of the red‐backed fairy wren (Malurus melanocephalus). *Evolution, 62*(12), 3117-3134.

McElroy, K., Beattie, K., Symonds, M. R. E., & Joseph, L. (2017). Mitogenomic and nuclear diversity in the Mulga Parrot of the Australian arid zone: cryptic subspecies and tests for selection. *Emu - Austral Ornithology, 118*(1), 22-35. doi:10.1080/01584197.2017.1411765

Mellick, R., Wilson, P. D., & Rossetto, M. (2014). Demographic history and niche conservatism of tropical rainforest trees separated along an altitudinal gradient of a biogeographic barrier. *Australian journal of botany, 62*(5), 438-450.

Moreau, C. S., Hugall, A. F., McDonald, K. R., Jamieson, B. G., & Moritz, C. (2015). An ancient divide in a contiguous rainforest: Endemic earthworms in the Australian Wet Tropics. *PloS one, 10*(9), e0136943.

Nicholls, J., & Austin, J. (2005). Phylogeography of an east Australian wet‐forest bird, the satin bowerbird (Ptilonorhynchus violaceus), derived from mtDNA, and its relationship to morphology. *Molecular Ecology, 14*(5), 1485-1496.

Nyári, Á. S., & Joseph, L. (2013). Comparative phylogeography of Australo-Papuan mangrove-restricted and mangrove-associated avifaunas. *Biological Journal of the Linnean Society, 109*(3), 574-598.

Peñalba, J. V., Mason, I. J., Schodde, R., Moritz, C., & Joseph, L. (2017). Characterizing divergence through three adjacent Australian avian transition zones. *Journal of Biogeography*.

Potter, S., Eldridge, M. D., Taggart, D. A., & Cooper, S. J. (2012). Multiple biogeographical barriers identified across the monsoon tropics of northern Australia: phylogeographic analysis of the brachyotis group of rock‐wallabies. *Molecular Ecology, 21*(9), 2254-2269.

Schneider, C., Cunningham, M., & Moritz, C. (1998). Comparative phylogeography and the history of endemic vertebrates in the Wet Tropics rainforests of Australia. *Molecular Ecology, 7*(4), 487-498.

Schweizer, M., Guentert, M., & Hertwig, S. T. (2013). Out of the Bassian province: historical biogeography of the Australasian platycercine parrots (Aves, Psittaciformes). *Zoologica Scripta, 42*(1), 13-27.

Toon, A., Hughes, J., & Joseph, L. (2010). Multilocus analysis of honeyeaters (Aves: Meliphagidae) highlights spatio‐temporal heterogeneity in the influence of biogeographic barriers in the Australian monsoonal zone. *Molecular Ecology, 19*(14), 2980-2994.
