# Supplementary material for: Pleistocene-dated biogeographic barriers drove divergence within the Australo-Papuan region in a sex-specific manner: an example in a widespread Australian songbird
Source: Heredity (Edinb). 2019 Mar 15;123(5):608–21. doi: 10.1038/s41437-019-0206-2 (PMC6972870; doi:10.1038/s41437-019-0206-2)
Supplement: Supplementary file 3 — Appendix S3 [file 41437_2019_206_MOESM3_ESM.doc]

**Appendix S3:** Nuclear intron PCR amplification and sequencing information

Sixty-nine individuals, representative of the entire range of the species, were sequenced for five autosomal and one Z-linked intron loci: aldolase B intron 4 (AB4), rhodopsin intron 2 (RI2), glyceraldehyde-3-phosphate dehydrogenase intron 11 (GAPDH11), transforming growth factor-β intron 5 (TGFβ2) using primers and conditions explained elsewhere (Appendix D of Pavlo*va et* al, 2014), the dopamine receptor D4 intron 3 and flanking exon sequences (DRD4) using primers DRD4-F14541 (5’-TACTGTGGCATGTTCCAAGG-3’) and DRD4-R15360 (5’-GGWGCAGGACTTGCAGAGAG-3’), and the Z-linked muscle-specific tyrosine kinase intron 4 (MUSK-I4) using primers MUSK-I4F2 (5’-AAATAACCCGACCACCTGTAAA-3’) and MUSK-I4R (5’-AGGCAGCAATTATCCTGCAC-3’). Amplification reactions contained 1 x KCl buffer (Fermentas), 1.5 mM MgCl_2_, 0.2 µM dNTPs, 0.4 µM primers and 0.01 unit/µL *Taq* polymerase to give a total reaction volume of 25 µL. PCRs were run using the following protocol: initial denaturation at 95°C for 15 mins, followed by 35 cycles of denaturation at 94°C for 30 sec, annealing at 55°C for 30 sec and extension at 72°C for 1 min, and concluded with final extension at 72°C for 10 min. Sequencing was conducted commercially using forward primers (at UK NERC Genepool sequencing facility, University of Edinburgh, UK or Macrogen, Korea). Sequence chromatograms were aligned and edited using Geneious Pro 7.0.6 (Kear*se et* al, 2012). Sequences containing indels or ambiguities were re-sequenced in the reverse direction.

**References**

Kearse M, Moir R, Wilson A, Stones-Havas S, Cheung M, Sturrock S *et al* (2012). Geneious Basic: an integrated and extendable desktop software platform for the organization and analysis of sequence data. *Bioinformatics* **28**(12)**:** 1647-1649.

Pavlova A, Selwood P, Harrisson KA, Murray N, Quin B, Menkhorst P *et al* (2014). Integrating phylogeography and morphometrics to assess conservation merits and inform conservation strategies for an endangered subspecies of a common bird species. *Biological conservation* **174:** 136-146.
