# Supplementary material for: Pleistocene-dated biogeographic barriers drove divergence within the Australo-Papuan region in a sex-specific manner: an example in a widespread Australian songbird
Source: Heredity (Edinb). 2019 Mar 15;123(5):608–21. doi: 10.1038/s41437-019-0206-2 (PMC6972870; doi:10.1038/s41437-019-0206-2)
Supplement: Supplementary file 4 — Appendix S4 [file 41437_2019_206_MOESM4_ESM.docx]

**­­Appendix S4:** TESS clustering analyses results

TESS calculates DIC values to convey the likelihood that a number of clusters (Kmax) represents the true number of clusters in a population: more likely Kmax values return smaller DIC values (Chen *et al*, 2007). Typically, in clustering analyses, the DIC reduces as Kmax increases until the true number of clusters (K) is reached. After K has been reached, the decrease in DIC at successively larger Kmax values plateaus. Here, the DIC values (mean and 95% confidence interval) of each run were calculated and plotted for each Kmax for TESS analyses of the length-variable marker (Figure S4.1a) and nuclear intron sequence (Figure S4.1b) datasets. ANOVAs confirmed that Kmax had a significant effect on run DIC value in the analyses of both datasets (*P*-values <0.001). Tukey’s tests were conducted *post-hoc* and identified that, for TESS analyses of both datasets, the DIC values were successively and significantly smaller for runs of successively larger Kmax values until Kmax was equal to five. The runs for Kmax = 6 did not have significantly smaller DIC values than the runs for Kmax = 5 (*P*-values >0.05). The DIC values of runs for Kmax = 5 were significantly but only slightly smaller than the DIC values of runs for Kmax = 4 for both the length-variable marker (Kmax 4 mean DIC value (SD) = 12270.0 (113.2) and Kmax 5 mean DIC value (SD) = 12227.0 (103.5)) and nuclear intron sequence data (Kmax 4 mean DIC value (SD) = 2510.5 (30.2) and Kmax 5 mean DIC value (SD) = 2490.7 (21.4)) (Figure S4.1a). We therefore constructed and present structure plots to depict genetic clustering within the species based on the length-variable marker (Figure S4.2a) and nuclear intron sequence datasets (Figure S4.2b) for Kmax = 4 and Kmax = 5. The cluster memberships presented in these plots represent the calculated cluster probabilities of the 10 replicates with the lowest DIC values of each K that were averaged using CLUMPP 1.1.2 (Jakobsson and Rosenberg, 2007).

For the length-variable marker data, the same four clusters were identified in the Kmax = 5 analyses as the Kmax = 4 analyses and maximum cluster membership of an individual to the fifth, additional cluster was 0.14 (Figure S4.2). The Kmax = 5 analyses were therefore concluded to provide no more resolution than the Kmax = 4 analyses and so the results of the Kmax = 4 analyses are presented in the main manuscript.

For the Kmax = 4 and Kmax = 5 analyses of the nuclear intron sequence data, three of the clusters identified map to three of the grey shrike-thrush subspecies: *harmonica* in east Australia, *strigata* in Tasmania and *rufiventris* in south-west/central Australia (Figure S4.2). The fourth cluster in the Kmax = 4 analysis mapped to *brunnea* in north-west Australia and *superciliosa* in Cape York Peninsula and New Guinea (Figure S4.2). Although the fourth and fifth clusters in the Kmax = 5 analyses map to *brunnea* and *superciliosa* individuals, the fourth cluster (purple) mainly maps to *superciliosa* individuals and the fifth cluster (green) mainly maps to *brunnea* individuals (ie. *brunnea* individuals had greater membership for the fifth cluster (green) than the fourth (purple) cluster and the opposite is true for *superciliosa* individuals) (Figure S4.2). In addition to having lower DIC values, the Kmax = 5 analyses provided more resolution than the Kmax = 4 analyses of the nuclear intron sequence data and are therefore presented in the main manuscript.

Chen C, Durand E, Forbes F, François O (2007). Bayesian clustering algorithms ascertaining spatial population structure: a new computer program and a comparison study. *Molecular Ecology Resources* **7**(5)**:** 747-756.

Jakobsson M, Rosenberg NA (2007). CLUMPP: a cluster matching and permutation program for dealing with label switching and multimodality in analysis of population structure. *Bioinformatics* **23**(14): 1801-1806.


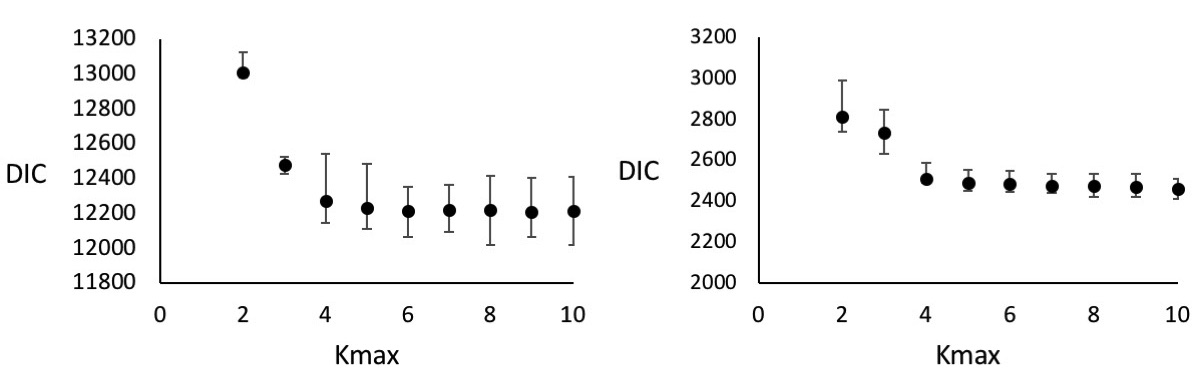


(a)

(b)

**Figure S4.1** DIC as a function of Kmax for the TESS analyses of the (a) length-variable marker and (b) nuclear intron sequence datasets. Dots and error bars, respectively, represent the mean and 95% confidence intervals of DIC values of the 100 runs of each Kmax.

**
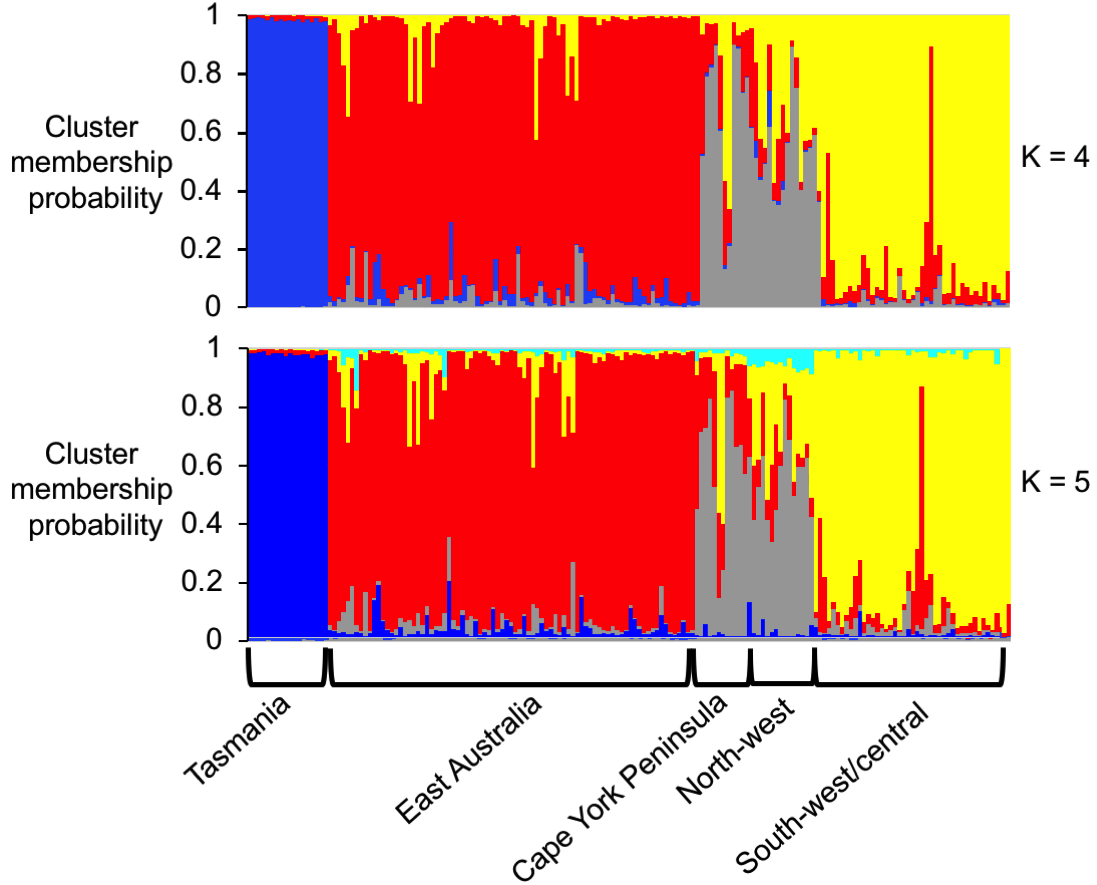
**

(a)

**
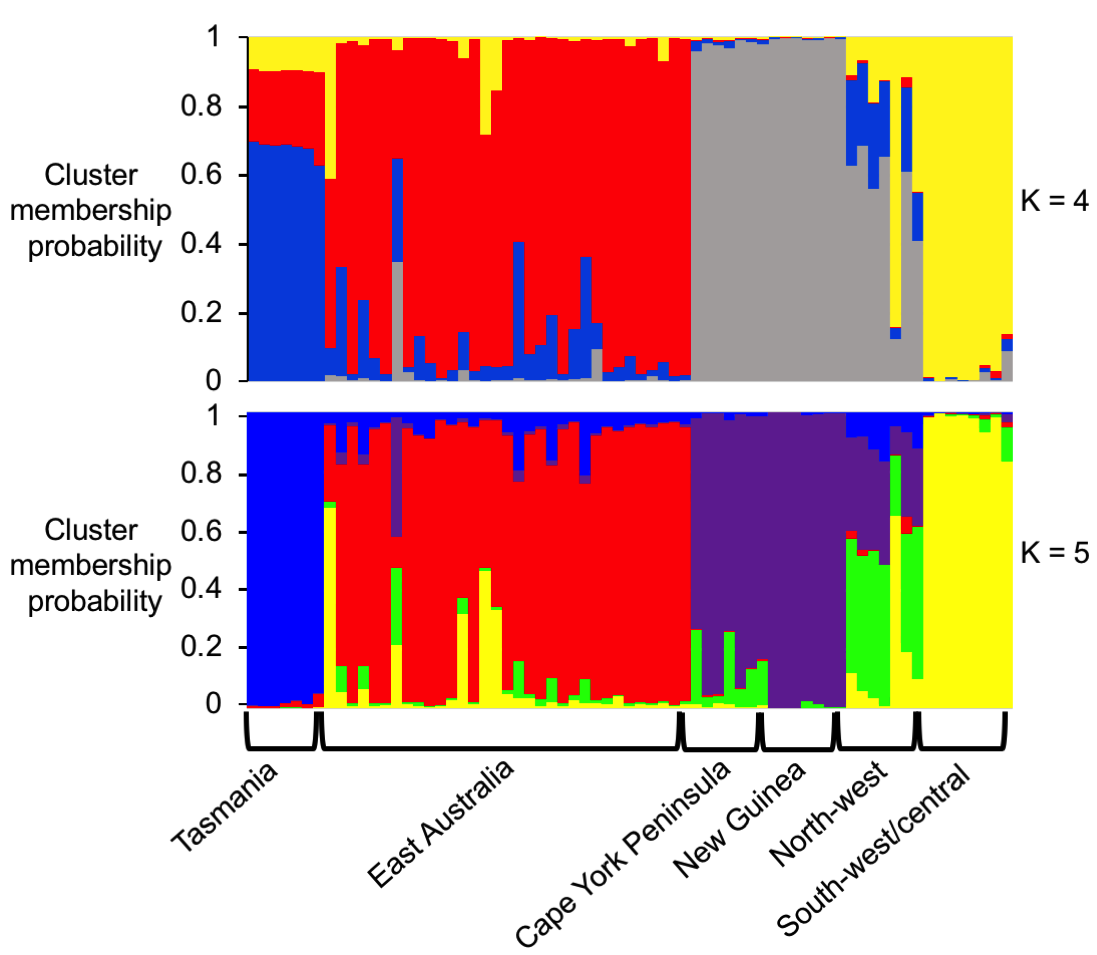
**

(b)

**Figure S4.2** Structure plots of the TESS cluster analyses results of the (a) length-variable loci and (b) nuclear intron sequence data for Kmax = 4 and Kmax = 5. Samples are grouped together along the x-axis by regions that have been defined based on the position of sampling locations in relation to contemporary and putative historical biogeographical barriers.
