# Supplementary material for: Pleistocene-dated biogeographic barriers drove divergence within the Australo-Papuan region in a sex-specific manner: an example in a widespread Australian songbird
Source: Heredity (Edinb). 2019 Mar 15;123(5):608–21. doi: 10.1038/s41437-019-0206-2 (PMC6972870; doi:10.1038/s41437-019-0206-2)
Supplement: Supplementary file 5 — Appendix S5 [file 41437_2019_206_MOESM5_ESM.doc]

Appendix S5: Nuclear intron haplotype networks


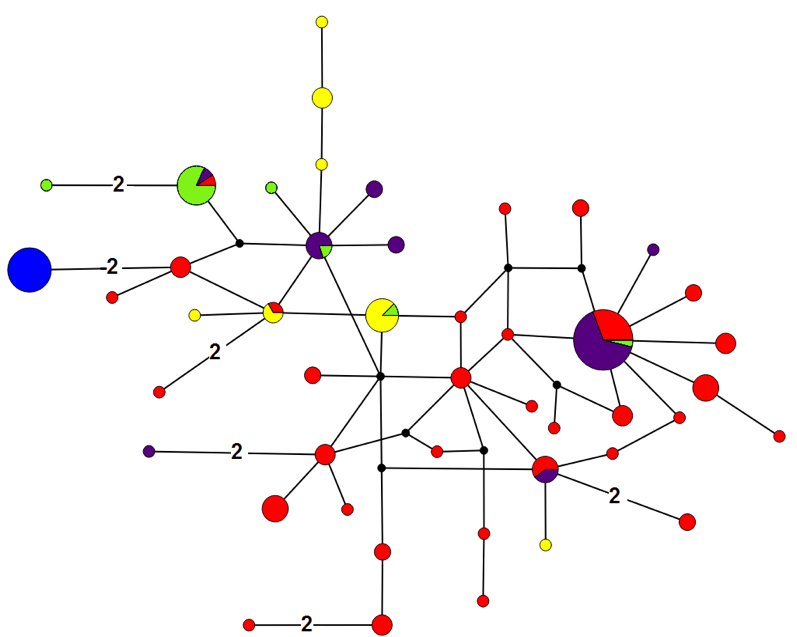


DRD4


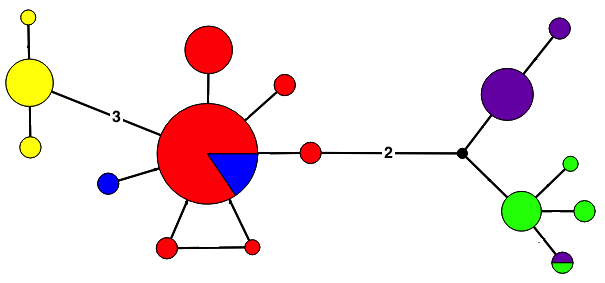


MUSK-I4


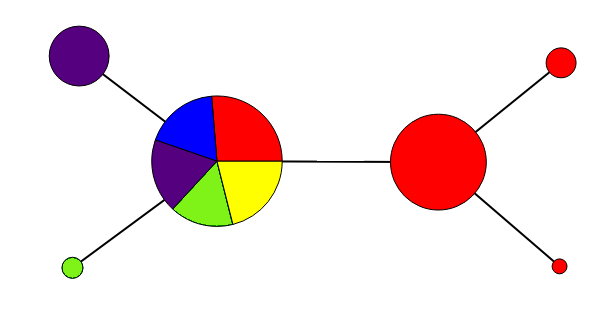


AB4


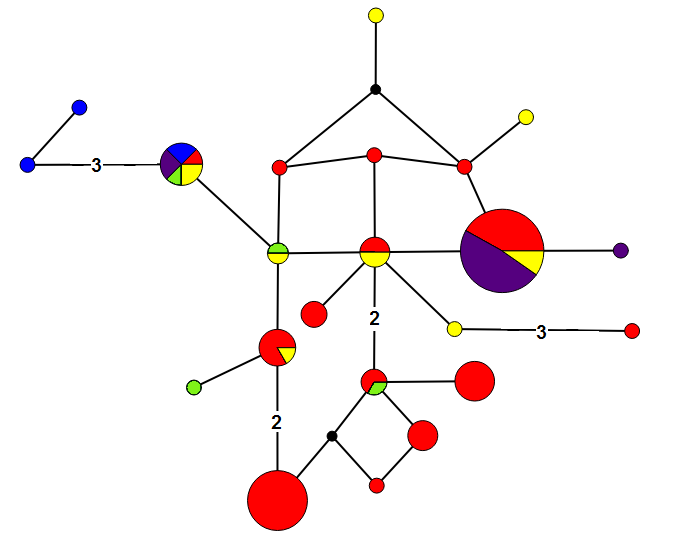


GAPDH11


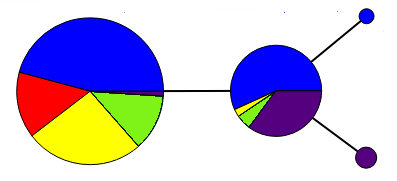


RI2


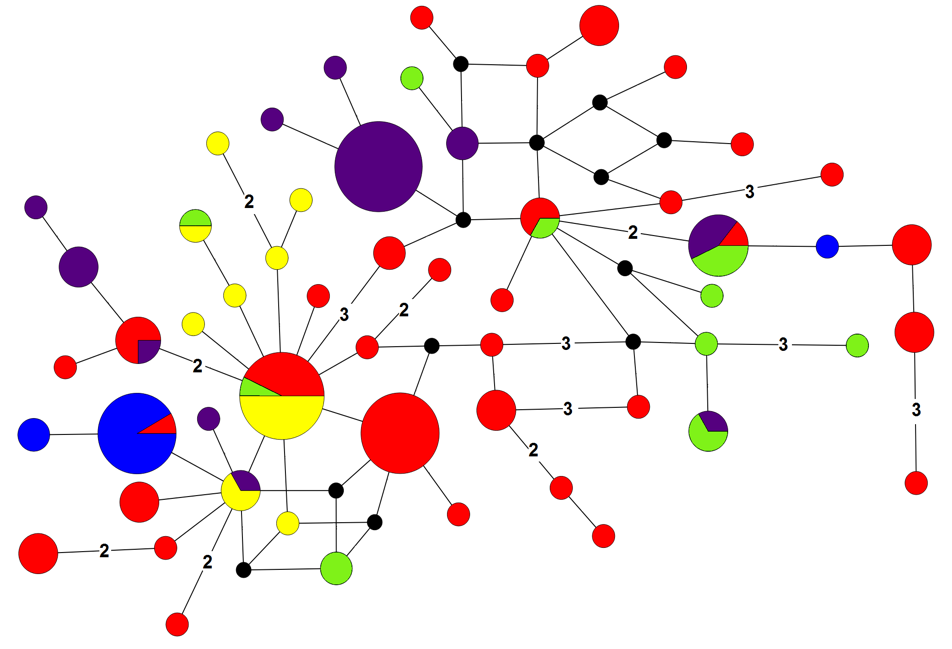


TGFβ2

**Figure S5.1** Nuclear intron median-joining haplotype networks. Circles represent unique haplotypes; they are proportional in size to the number of sampled individuals with that haplotype and coloured according to the proportion of those individuals from each of the mitochondrial clades, which have boundaries that tightly correlate with subspecies boundaries (coloured as in the figures in the main manuscript). Small black circles represent haplotypes that were inferred during the analyses but not sampled. Numbers bisecting the lines indicate the number of base differences between the haplotypes that those lines connect. Unmarked lines between haplotypes indicate differences between haplotypes of one base.
