# Supplementary material for: Pleistocene-dated biogeographic barriers drove divergence within the Australo-Papuan region in a sex-specific manner: an example in a widespread Australian songbird
Source: Heredity (Edinb). 2019 Mar 15;123(5):608–21. doi: 10.1038/s41437-019-0206-2 (PMC6972870; doi:10.1038/s41437-019-0206-2)
Supplement: Supplementary file 6 — Appendix S6 [file 41437_2019_206_MOESM6_ESM.doc]

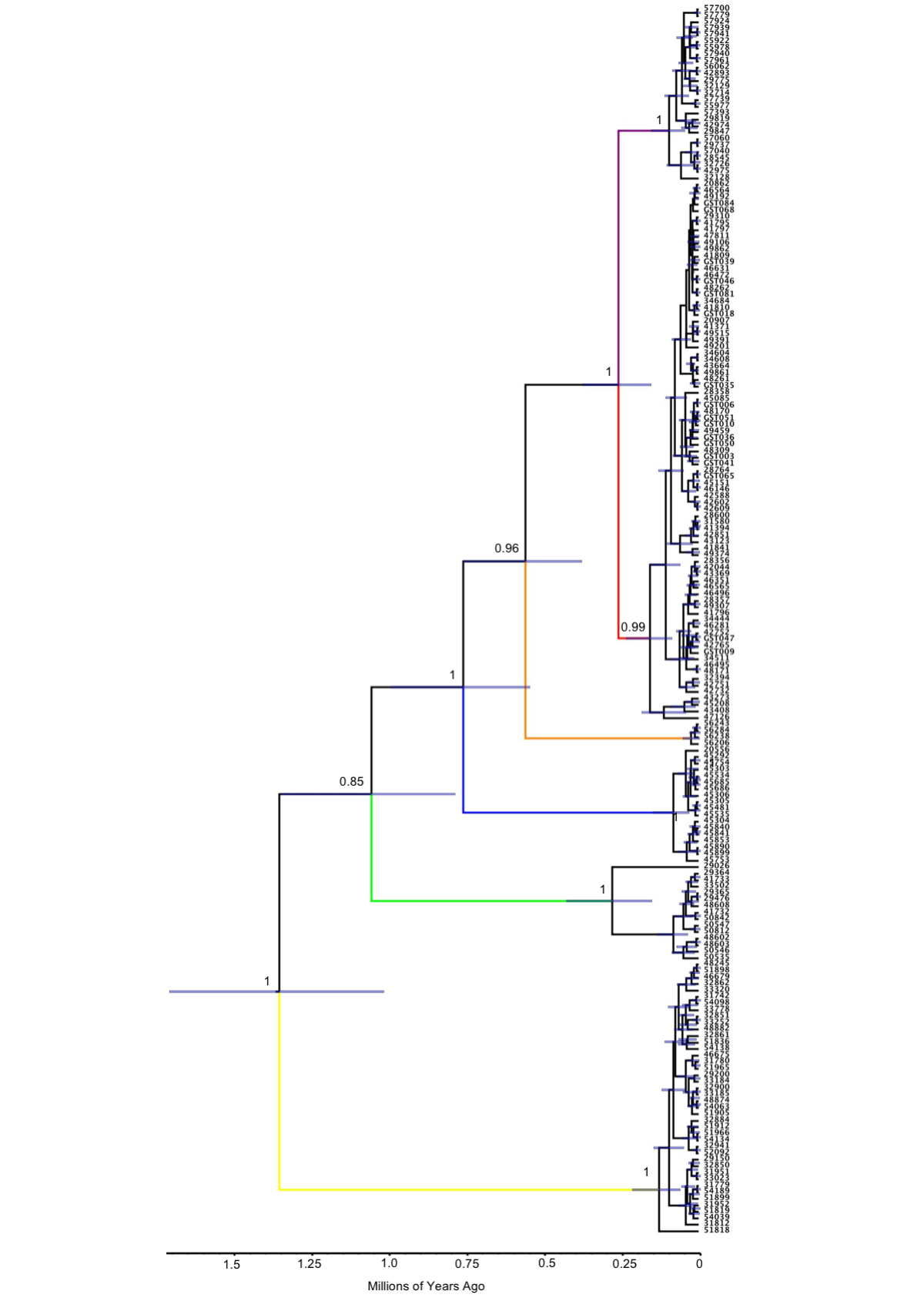
**Appendix S6:** Detailed ND2 phylogeny

**Figure S6.1** Detailed time-calibrated maximum clade credibility tree derived from ND2 data. Major nodes are labelled with their support (posterior probabilities). Node bars indicate 95% highest probability density ranges for divergence time estimates.
