# Supplementary material for: Pleistocene-dated biogeographic barriers drove divergence within the Australo-Papuan region in a sex-specific manner: an example in a widespread Australian songbird
Source: Heredity (Edinb). 2019 Mar 15;123(5):608–21. doi: 10.1038/s41437-019-0206-2 (PMC6972870; doi:10.1038/s41437-019-0206-2)
Supplement: Supplementary file 7 — Appendix S7 [file 41437_2019_206_MOESM7_ESM.doc]

**Appendix S7** MIGRATE-N results

**Table S7.1** Migration rates among Australian mainland populations of grey shrike-thrush. Estimates of migration scaled by mutation rates (
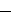
, effective population size (θ) scaled by mutation rates and migration rates in number of individuals (n) per generation are listed together with confidence intervals. Where confidence intervals overlap with zero, values are listed as non-significant (ns).

| **Migration direction** | 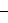 **_A🡪B_** | **θ_B_** | **Migration (n/generation)** |
| --- | --- | --- | --- |
| East Australian to Cape York Peninsula | 0.54 (0.18-0.91) | 4.90 (3.13-6.74) | 0.66 (0.22-1.11) |
| East Australian to north-west | ns | 4.29 (2.84-5.92) | Ns |
| East Australian to south-west and central | 0.40 (0.11-0.71) | 4.26 (2.86-5.76) | 0.42 (0.12-0.76) |
| Cape York Peninsula to east Australian | 0.39 (0.11-0.71) | 4.29 (2.86-5.98) | 0.42 (0.11-0.76) |
| Cape York Peninsula to north-west | ns | 4.29 (2.84-5.92) | ns |
| Cape York Peninsula to south-west and central | ns | 4.26 (2.86-5.76) | ns |
| North-west to east Australian | 0.53 (0.17-0.93) | 4.29 (2.86-5.98) | 0.57 (0.18-1.00) |
| North-west to Cape York Peninsula | 0.62 (0.21-1.07) | 4.90 (3.13-6.74) | 0.76 (0.26-1.31) |
| North-west to south-west and central | ns | 4.26 (2.86-5.76) | ns |
| South-west and central to east Australian | 0.58 (0.18-0.99) | 4.29 (2.86-5.98) | 0.62 (0.20-1.06) |
| South-west and central to Cape York Peninsula | ns | 4.90 (3.13-6.74) | ns |
| South-west and central to north-west | 0.45 (0.12-0.82) | 4.29 (2.84-5.92) | 0.36 (0.13-0.88) |
